# Leukocyte Bim deficiency does not impact atherogenesis in *ldlr*^−/−^ mice, despite a pronounced induction of autoimmune inflammation

**DOI:** 10.1038/s41598-017-02771-4

**Published:** 2017-06-08

**Authors:** Lieve Temmerman, Marijke M. Westra, Ilze Bot, Bart J. M. van Vlijmen, Niek van Bree, Martine Bot, Kim L. L. Habets, Tom G. H. Keulers, Johan van der Vlag, Thomas G. Cotter, Theo J. C. van Berkel, Erik A. L. Biessen

**Affiliations:** 1 Experimental Vascular Pathology, Department of Pathology, Cardiovascular Research Institute Maastricht, Maastricht University, Maastricht, The Netherlands; 2 Division of Biopharmaceutics, Leiden Academic Centre for Drug Research, Leiden University, Leiden, The Netherlands; 3 Department of Thrombosis Hemostasis, Leiden University Medical Centre, Leiden, The Netherlands; 4 Department of Radiotherapy (MAASTRO)/GROW, School for Developmental Biology and Oncology, Maastricht University, Maastricht, The Netherlands; 5 Department of Nephrology, Radboud University Medical Center, Nijmegen, The Netherlands; 6 Cell Development and Disease Laboratory, Department of Biochemistry, Biosciences Research Institute, University College Cork, Cork, Ireland; 7 Institute for Molecular Cardiovascular Research (IMCAR), University Hospital RWTH, Aachen, Germany

## Abstract

Proapoptotic Bcl-2 family member Bim is particularly relevant for deletion of autoreactive and activated T and B cells, implicating Bim in autoimmunity. As atherosclerosis is a chronic inflammatory process with features of autoimmune disease, we investigated the impact of hematopoietic Bim deficiency on plaque formation and parameters of plaque stability. *Bim* ^−/−^ or wild type bone marrow transplanted *ldlr* ^−/−^ mice were fed a Western type diet (WTD) for 5 or 10 weeks, after which they were immunophenotyped and atherosclerotic lesions were analyzed. *Bim* ^−/−^ transplanted mice displayed splenomegaly and overt lymphocytosis. CD4^+^ and CD8^+^ T cells were more activated (increased CD69 and CD71 expression, increased interferon gamma production). B cells were elevated by 147%, with a shift towards the pro-atherogenic IgG-producing B2 cell phenotype, resulting in a doubling of anti-oxLDL IgG1 antibody titers in serum of *bim* ^−/−^ mice. *Bim* ^−/−^ mice displayed massive intraplaque accumulation of Ig complexes and of lesional T cells, although this did not translate in changes in plaque size or stability features (apoptotic cell and macrophage content). The surprising lack in plaque phenotype despite the profound pro-atherogenic immune effects may be attributable to the sharp reduction of serum cholesterol levels in WTD fed *bim* ^−/−^ mice.

## Introduction

Atherosclerosis is a chronic inflammatory disease of the large vessels characterized by lipid accumulation in the intimal wall^1^. Oxidized low density lipoprotein (LDL) functions as an autoantigen^2^, and a “vascular autoimmunosome” has recently been identified stressing the importance of autoimmunity in vascular injury and plaque development^3^. As the disease progresses, apoptosis in the vascular wall gradually increases^4^. In early atherogenesis apoptosis is regarded to be a beneficial factor as it dampens plaque inflammation and limits lesion expansion^5, 6^. At later stages of atherosclerosis however, clearance of apoptotic cells is insufficient^7^, and the ensuing secondary necrosis may here promote necrotic core formation and inflammation, both hallmarks of plaque destabilization.

The Bcl-2 family of pro- and anti-apoptotic proteins regulates apoptosis induced by cellular stressors such as DNA damage, UV radiation and oxidative stres^8^. Proteins of this family share one to four Bcl-2 homology (BH) domains^8^. *In vivo* studies in atherosclerotic mouse models have established a role for various Bcl-2 family members in apoptosis of atherosclerotic lesion macrophages^9, 10^. Bim (Bcl-2 interacting mediator of cell death) is a BH3-only pro-apoptotic protein of the Bcl-2 family^11, 12^ and, like other BH3-only proteins, binds to anti-apoptotic Bcl-2 family members thereby initiating apoptosis^13^. Studies in Bim deficient mice have revealed crucial functions of Bim in leukocyte homeostasis. Bim deficient granulocytes and lymphocytes are less sensitive to apoptosis induced by cytokine deprivation or various pro-apoptotic stimuli^14, 15^. Moreover, Bim deficient mice display leukocytosis, with markedly elevated B and T cell numbers in circulation, spleen and thymus, and more circulating monocytes and granulocytes^14^. Bim was seen to be necessary for appropriate control and termination of immune responses^16^ and accordingly, its deficiency resulted in autoimmunity and lymphadenopathy due to defective removal of autoreactive T and B cells^14, 17, 18^.

Given its critical function in leukocyte homeostasis and autoimmunity control, a role for this protein in the pathogenesis of atherosclerosis is anticipated. Therefore, in the present study we have investigated the role of Bim regulated leukocyte apoptosis in atherosclerosis-prone *ldlr* ^−/−^ mice. Our study shows that hematopoietic Bim deficiency in *ldlr* ^−/−^ mice results in increased atherosclerotic lesion T cell content and massive immunoglobulin deposition as well as increased circulating T and B cells and high levels of anti-oxidized LDL autoantibodies. Furthermore, we demonstrate that loss of leukocyte Bim interferes with lipid metabolism.

## Results

### Bim^−/−^ BM transplanted mice have splenomegaly and increased levels of circulating lymphocytes

**Figure 1:**
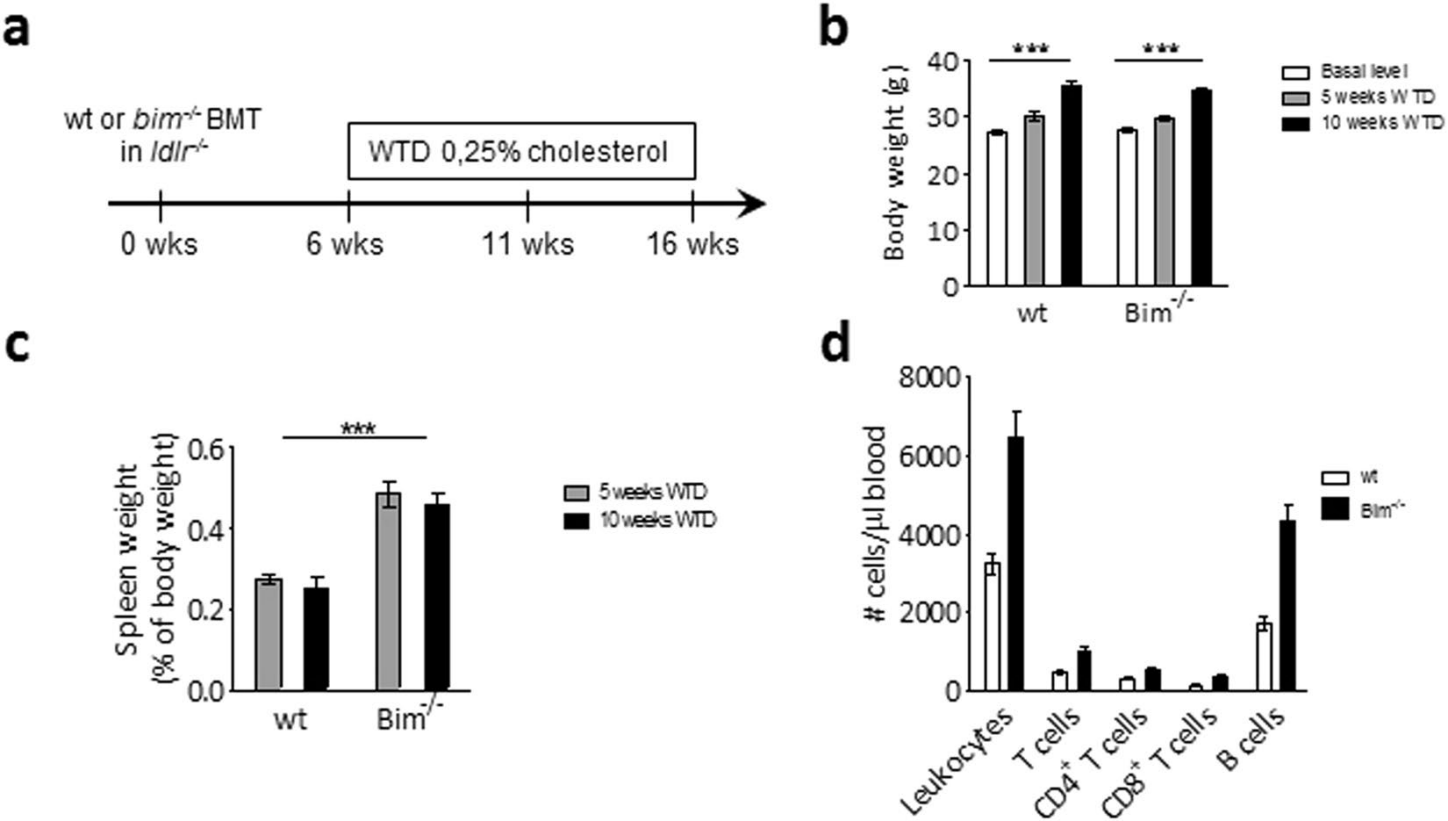
*Bim* ^−/−^ chimeric mice have splenomegaly and lymphocytosis. (**a**) Lethally irradiated *ldlr* ^−/−^ mice were reconstituted with wt or *bim* ^−/−^ bone marrow, and after 6 weeks recovery, put on a Western Type Diet containing 0.25% cholesterol for 5 (n = 7) or 10 weeks (n = 12). (**b**) Body weight of wt and *bim* ^−/−^ chimeric mice. Data is presented as mean ± SEM. ***p < 0.001 in two-way ANOVA. (**c**) Relative spleen weight of wt and *bim* ^−/−^ chimeric mice at sacrifice. Data is presented as mean ± SEM. ***p < 0.001 in two-way ANOVA. (**d**) TruCount tubes were used for quantitative analysis of leukocyte subsets in blood of wt and *bim* ^−/−^ chimeric mice after 5 weeks of WTD. Data is presented as mean ± SEM. p < 0.001 between wt and *bim* ^−/−^ groups in two-way ANOVA.

The role of leukocyte Bim expression in atherogenesis was studied in chimeras generated by transplanting bone marrow (BM) from *bim* ^−/−^ and wt littermates to irradiated *ldr* ^−/−^ recipients. After recovery, all mice were fed a Western-type diet containing 0.25% cholesterol (WTD) for 5 (initial atherogenesis) or 10 (advanced atherosclerosis) weeks (Fig. 1a). Wt and *bim* ^−/−^ chimeric mice (from here on wt and *bim* ^−/−^ mice) showed progressive but equivalent weight gain (Fig. 1b). After 5 weeks of WTD, neutrophil counts in spleen as well as in circulation, were unaffected by the loss of Bim (Sup. Fig. 1). While circulating monocyte numbers were unaffected, those of spleen were slightly reduced in *bim* ^−/−^ mice. Furthermore both in spleen and in blood we observed a shift towards non-classical “inflammatory” Ly6C^low^ monocytes, compatible with dampened WTD associated Ly6C^high^ monocytosis (Sup. Fig. 1). Both at 5 and at 10 weeks of WTD however, *bim* ^−/−^ mice suffered from splenomegaly (1.8 fold increase in relative weight, Fig. 1c) and leukocytosis (Fig. 1d). The latter was attributable to expansion of total T cells - both CD4^+^ and CD8^+^ - and B cells in blood of the *bim* ^−/−^ mice (Fig. 1d).

### Apoptotic cell death is affected by loss of leukocyte Bim

**Figure 2:**
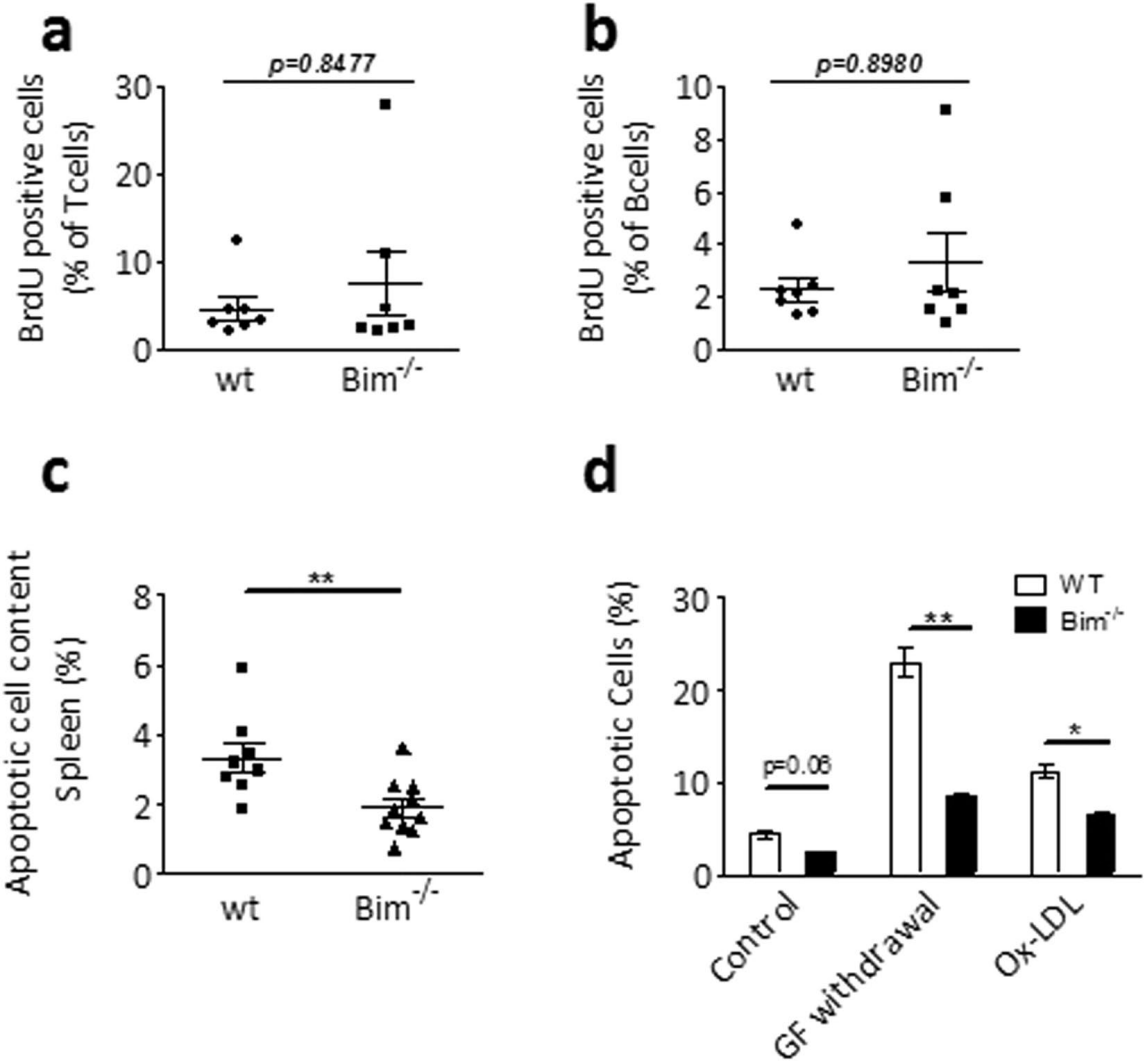
*Bim* ^−/−^ leukocytes are more resistant towards apoptosis. (**a**,**b**) After 5 weeks of WTD, wt and *bim* ^−/−^ chimeric mice were injected with BrdU 24 and 12 hrs before sacrifice to quantify proliferating cells. BrdU incorporation in T (**a**) and B (**b**) cell subsets was measured by flow cytometry. Mean ± SEM are indicated. (**c**) Cyrosections of wt and *bim* ^−/−^ spleens after 10 weeks of WTD were analyzed by TUNEL staining to quantify apoptotic cells (n = 8 for wt, n = 10 for *bim* ^−/−^). Data is presented as mean ± SEM. **p < 0.01 in student’s *t* Test. (**d**) wt and *bim* ^−/−^ bone marrow derived macrophages (n = 5) were exposed to different apoptotic stimuli (growth factor withdrawal or oxLDL 40 μg/ml) and percentage of apoptotic cells was quantified by flow cytometry based on AnnexinV and Propidium Iodide signals. Body weight of wt and *bim* ^−/−^ chimeric mice. Data is presented as mean ± SEM. *p < 0.05, **p < 0.01 in Mann-Whitney *U* Test.

24 hours before sacrifice, mice were injected with BrdU, and we evaluated BrdU incorporation in splenocytes by flow cytometry. The Bim deficiency associated splenomegaly could not be explained by increased proliferation, as there were no differences between wt and *bim* ^−/−^ mice in BrdU-positive T and B cells (Fig. 2a,b). However, apoptotic cell content in *bim* ^−/−^ spleens *in vivo* was significantly lower than in wt spleens (1.8 ± 0.3 vs. 3.3 ± 0.4% of total splenocytes respectively, p < 0.01, Fig. 2c). Bim’s proapoptotic activity was further confirmed in *bim* ^−/−^ bone marrow derived macrophages (BMDM), which showed decreased sensitivity to apoptotic cell death at baseline, and in response to known proapoptotic stimuli (i.e. growth factor withdrawal and oxidized LDL) (Fig. 2d). Taken together, these data clearly demonstrate that changes in leukocyte numbers in *bim* ^−/−^ atherosclerotic mice are a consequence of increased cell survival due to the loss of pro-apoptotic Bim.

### Loss of Bim favors a Th1 cytolytic immune profile

**Figure 3:**
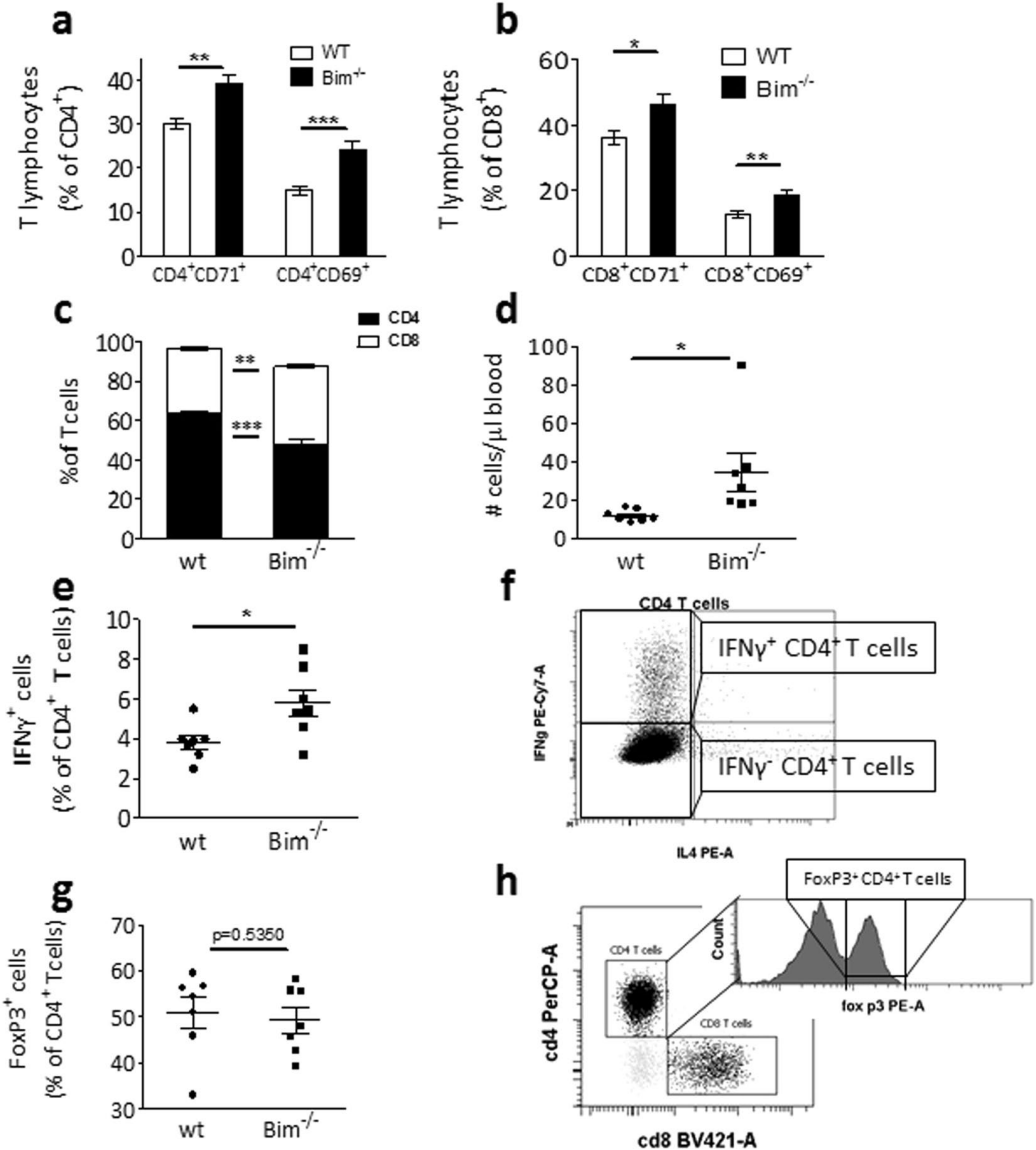
*Bim* ^−/−^ T cells are more activated. (**a**,**b**) After 10 weeks of WTD, CD69 and CD71 expression on CD4^+^ and CD8^+^ T cells, respectively, were quantified by flow cytometry (n = 12). (**c**) The CD4^+^/CD8^+^ T cell ratio is disturbed in *bim* ^−/−^ chimeric mice. Flow cytometry measurements on blood after 5 weeks of WTD (n = 7). (**d**) *Bim* ^−/−^ chimeric mice have more Tbet^+^ CD4^+^ T cells. TruCount flow cytometry measurements after 5 weeks of WTD (n = 7). (**e**) Wt and *bim* ^−/−^ splenocytes were harvested after 5 weeks of WTD, stimulated *in vitro* with PMA and ionomycin and IFNγ production in T cells was quantified using flow cytometry (n = 7). (**f**) Gatings for the IFNγ positive cells quantified in (**e**). (**g**) Regulatory T cell populations are similar between *bim* ^−/−^ and wt chimeric mice. Flow cytometry measurements on bone marrow after 5 weeks of WTD (n = 7). (**h**) Gatings for the FoxP3^+^ Treg cells cells quantified in (**g**). Data is presented as mean ± SEM. *p < 0.05, **p < 0.01, ***p < 0.001 in Mann-Whitney *U* Tests.

Related to Bim’s role in lymphocyte cell survival, it has also been associated with autoimmunity^14^. We therefore evaluated the consequences of hematopoietic Bim deficiency on adaptive immune cells. CD4^+^ and CD8^+^ T cell subsets were found to be more activated in *bim* ^−/−^ mice in comparison with wt mice as judged from the enhanced expression of activation markers CD69 and CD71 (p < 0.05, Fig. 3a,b). In addition, while all T cell subsets increased in absolute numbers, the deletion of Bim resulted in a clear shift towards more CD8^+^ T cells (Fig. 3c). Moreover T cell polarization was skewed towards Th1, as evidenced by a higher proportion of Tbet^+^ CD4^+^ T cells in blood of *bim* ^−/−^ mice as well as a higher production of IFNγ by CD4^+^ T cells in response to *ex vivo* PMA stimulation (Fig. 3d,e,f). Contrarily, wt and *bim* ^−/−^ PMA-stimulated CD4^+^ T cells did not show differences in IL4 production (Sup. Fig. 2a,b) and regulatory T cells were unaffected by loss of Bim (Fig. 3g,h). In addition, we sorted CD4^+^ T cells from wt and *bim* ^−/−^ chimeric mice. Pure, sorted CD4^+^ T cells from wt and *bim* ^−/−^ mice after 5 weeks on WTD were *in vitro* stimulated with PMA/ionomycin and expression levels of IL-10, IL-17, IFNγ and TNFα were measured by real-time PCR. *Bim* ^−/−^ derived CD4^+^ T cells tended to show higher IFNγ expression, confirming our intracellular expression data. IL-10 expression was unchanged, and TNFα as well as IL-17 expression were undetectable in both wt and *bim* ^−/−^ derived CD4^+^ T cells (Sup. Fig. 2c–e). Bim deficiency in atherosclerotic mice thus creates a more Th1/CD8^+^ oriented cellular T cell response without significantly altering other T cell reponses.

### Leukocyte Bim deficiency affects the humoral immune response in *ldlr*^−/−^ mice

**Figure 4:**
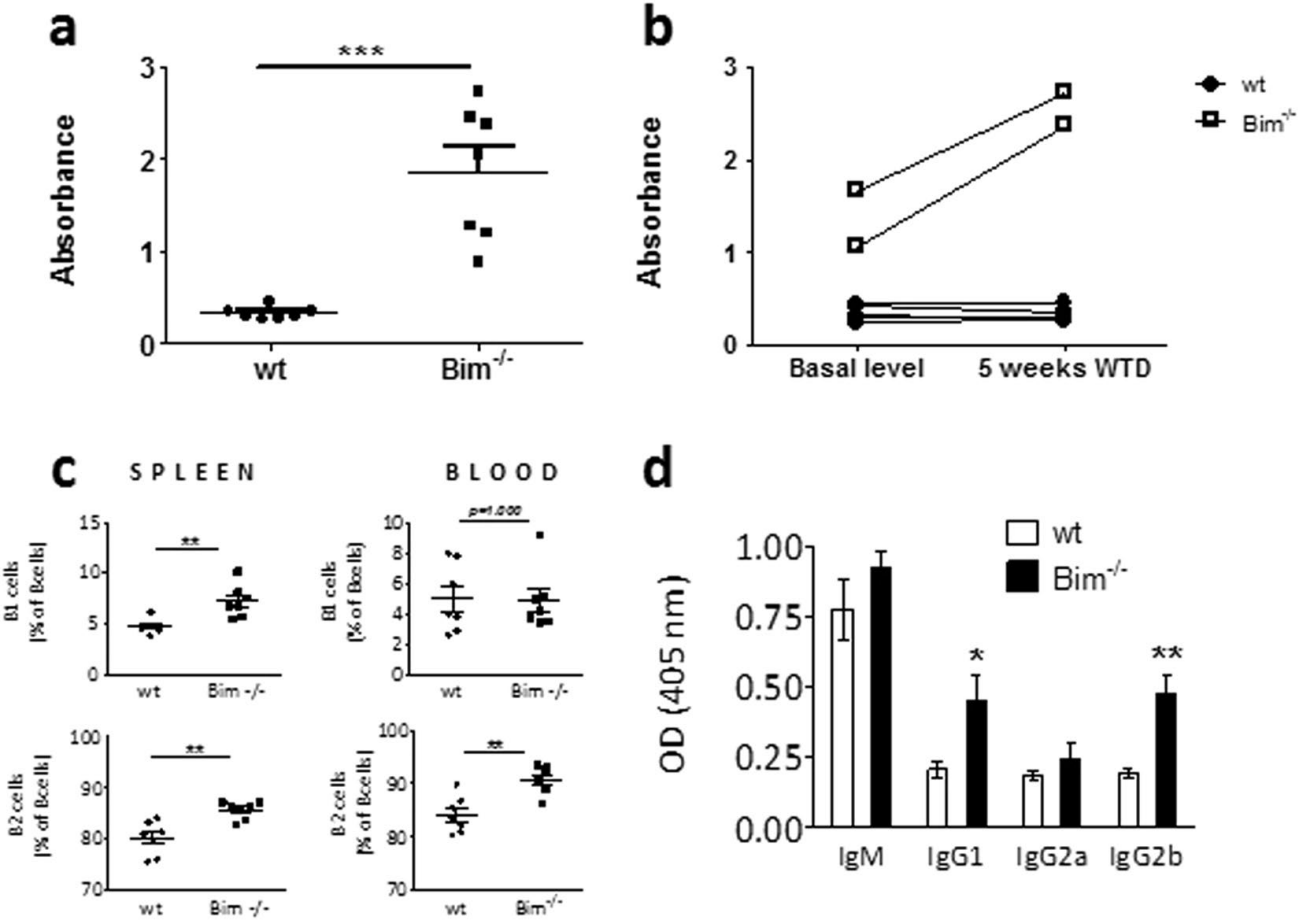
Bim deficiency alters the humoral response in atherosclerotic mice. (**a**) After 5 weeks of WTD, anti-dsDNA autoantibodies in sera of wt and *bim* ^−/−^ chimeric mice (n = 7) were quantified by ELISA. (**b**) Paired anti-dsDNA autoantibody determination in sera before start of WTD and after 5 weeks of WTD in 5 wt and 2 *bim* ^−/−^ chimeric mice. 2-way ANOVA test shows highly significant effects of both the *bim* ^−/−^ phenotype as well as the induction of the diet (p < 0.0001). (**c**) B1 and B2 cells in the blood and spleen of wt or *bim* ^−/−^ chimeric mice after 5 weeks of WTD were defined as CD19^+^, CD220^+^, CD11b^+^ (B1) and CD19^+^, CD220^+^, CD5^−^, CD11b^−^ (B2) respectively using flow cytometry (n = 7). (**d**) oxLDL specific antibody titers were measured by ELISA in sera of wt or *bim* ^−/−^ chimeric mice after 10 weeks of WTD (n = 12). Data is presented as mean ± SEM. *p < 0.05, **p < 0.01, ***p < 0.001.

Apart from its impact on T cell homeostasis, loss of Bim was reported to be associated with impaired deletion of autoreactive B cells. The resulting accumulation of these cells and derived autoreactive antibodies *in vivo* can promote autoimmunity^18^. An important indicator that autoimmune processes are involved is the appearance of anti-dsDNA autoantibodies in the blood, which were indeed observed in *bim* ^−/−^ mice and increased upon WTD feeding (Fig. 4a,b). Moreover, *bim* ^−/−^ mice showed a marked expansion of the B2 subset in blood and spleen (Fig. 4c). This was not the case for circulating B1 cells, while splenic B1 cells were also increased, but to a much lower extend than the B2 cell population. B2 cells are responsible for the production of antigen-specific IgG antibodies. OxLDL is an established autoantigen in atherosclerosis and IgG type anti-OxLDL autoantibodies are considered pro-atherogenic^19, 20^. We therefore measured OxLDL directed autoantibody titers in serum after 10 weeks of WTD. IgG1 and IgG2b anti-OxLDL antibodies were increased by more than two-fold (p < 0.05, Fig. 4d) in *bim* ^−/−^ mice compared to controls; no differences were detected in IgM and IgG2a anti-OxLDL autoantibody titers.

### Atherosclerotic lesions in bim^−/−^ mice are hallmarked by T cell infiltration and immunoglobulin deposits

**Figure 5:**
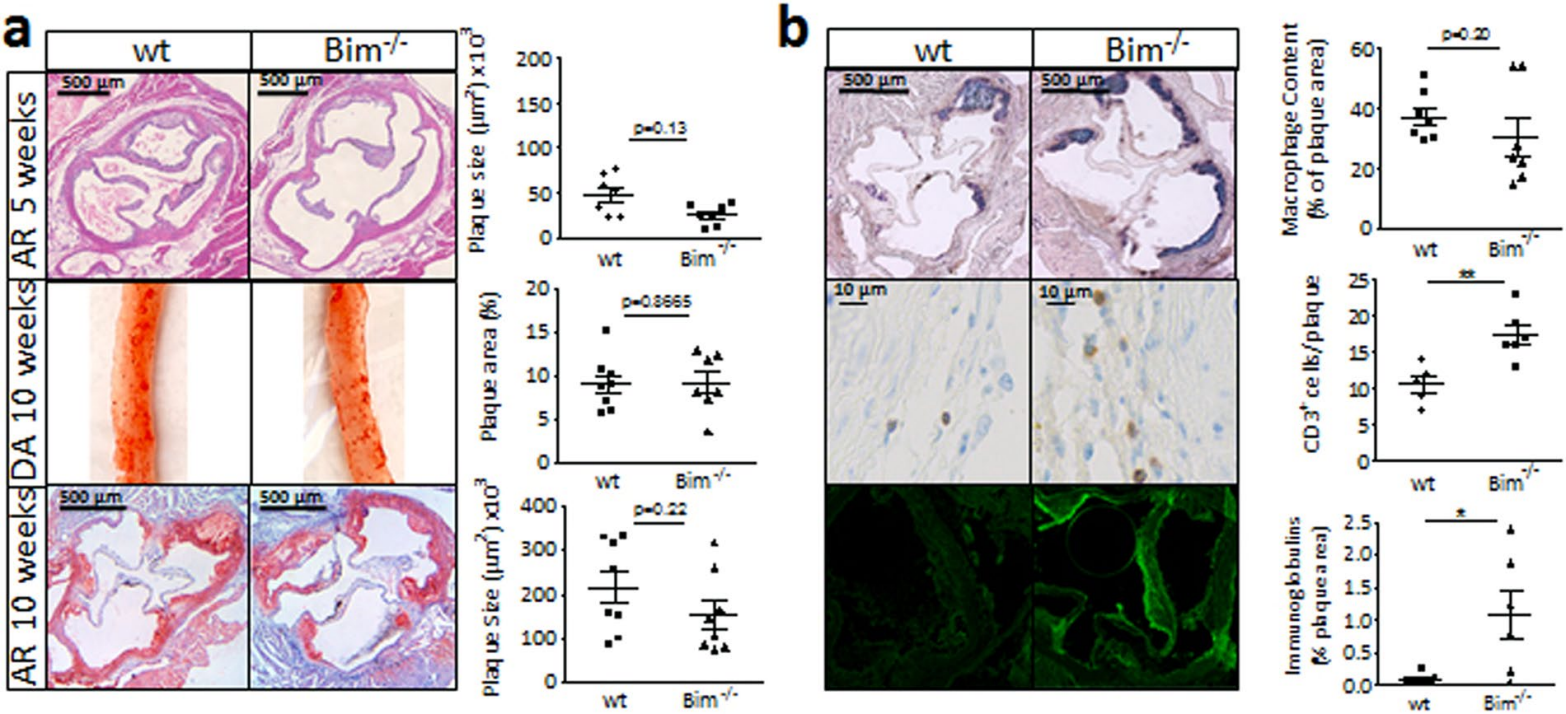
Plaques of *bim* ^−/−^ chimeras are marked by T cell accumulation and large Ig deposits. (**a**) Representative pictures of aortic root sections after 5 weeks and 10 weeks of WTD (top and bottom panel respectively), and descending aorta after 10 weeks of WTD (middle panel) reveal no differences in plaque size between wt and *bim* ^−/−^ chimeric mice. N = 7 (5 weeks WTD), n = 12 (10 weeks WTD). Data is shown as mean ± SEM. (**b**) Phenotypic analysis of atherosclerotic plaque composition in wt and *bim* ^−/−^ chimeric mice after 10 weeks of WTD (n = 12). Macrophage content was quantified by MOMA staining (top panel, blue), CD3 staining was used to identify T cells (middle panel, brown), and Ig complexes were visualized using FITC-labeled anti-mouse Ig. Data is presented as mean ± SEM. *p < 0.05, **p < 0.01.

The pronounced Th1 shift, combined with the increase in IgG autoantibodies, effects that both are considered pro-atherogenic, led us to expect a more severe plaque phenotype in *bim* ^−/−^ chimeras. Much to our surprise, we did not observe any significant differences in lesion size in aortic root between groups at atherosclerosis onset (5 weeks WTD) or in more advanced plaques (10 weeks WTD) (Fig. 5a top and bottom panels respectively); if anything plaques even tended to be smaller. In agreement, *en face* analysis of the descending aorta after 10 weeks of WTD showed no differences in atherosclerotic lesion area as well (Fig. 5a middle panels). Regarding composition, lesional MOMA-2 positive macrophage content in *bim* ^−/−^ did not differ from that in wt mice (Fig. 5b, top panels). Moreover, TUNEL staining of 10 weeks WTD aortic root sections as well as cleaved caspase 3 staining of 5 weeks WTD aortic root sections revealed no differences in lesional apoptotic cell content between wt and *bim* ^−/−^ mice (Sup. Fig. 3a,b).

As this was a somewhat surprising result, considering our earlier observed decreased sensitivity to apoptosis in spleen and in BMDMs, we assessed the functional profile of *bim* ^−/−^ macrophages. Hereto wt and *bim* ^−/−^ BMDMs were stimulated with LPS or IL4 to obtain M1- and M2-polarized macrophages respectively and gene expression was analyzed by real-time PCR. Apart from a slightly higher IL6 expression after LPS stimulation, other M1 cytokines (IL10, TNFα) and M2 cytokines (Mannose Receptor, Fizz1, YM1) did not change between wt and *bim* ^−/−^ BMDMs (Sup. Fig. 3c,d,e). Apoptotic content in the plaque is the resultant of the apoptosis rate (which could be reduced in *bim* ^−/−^ macrophages) and the capacity of plaque macrophages to efficiently clear away dead cells. Therefore, we analyzed efferocytosis in wt and *bim* ^−/−^ BMDMs. *Bim* ^−/−^ BMDMs appeared to be less effective in efferocytosis (p = 0.05; Sup. Fig. 3f,g).

In keeping with elevated T cell levels in circulation and lymphoid organs, T cell content in the atherosclerotic lesions (intima and adventitia) was increased by 51% from 10.1 ± 1.2 per section in wt mice to 15.3 ± 1.6 per section in *bim* ^−/−^ mice (p < 0.05, Fig. 5b, middle panels). Importantly, staining for deposits of total immunoglobulins (Ig) in lesions revealed the striking presence of Ig complexes in lesions of *bim* ^−/−^ chimeras but not littermate controls (0.10 ± 0.04% versus 1.08 ± 0.04%; p < 0.05, Fig. 4b, bottom panels). In summary, lesions in *bim* ^−/−^ animals did not differ in size or apoptotic cell content, although they demonstrated clear signs of increased cellular T cell as well as humoral (Ig complexes) immunity.

### Loss of leukocyte Bim reduces high fat diet-induced hyperlipidemia

**Figure 6:**
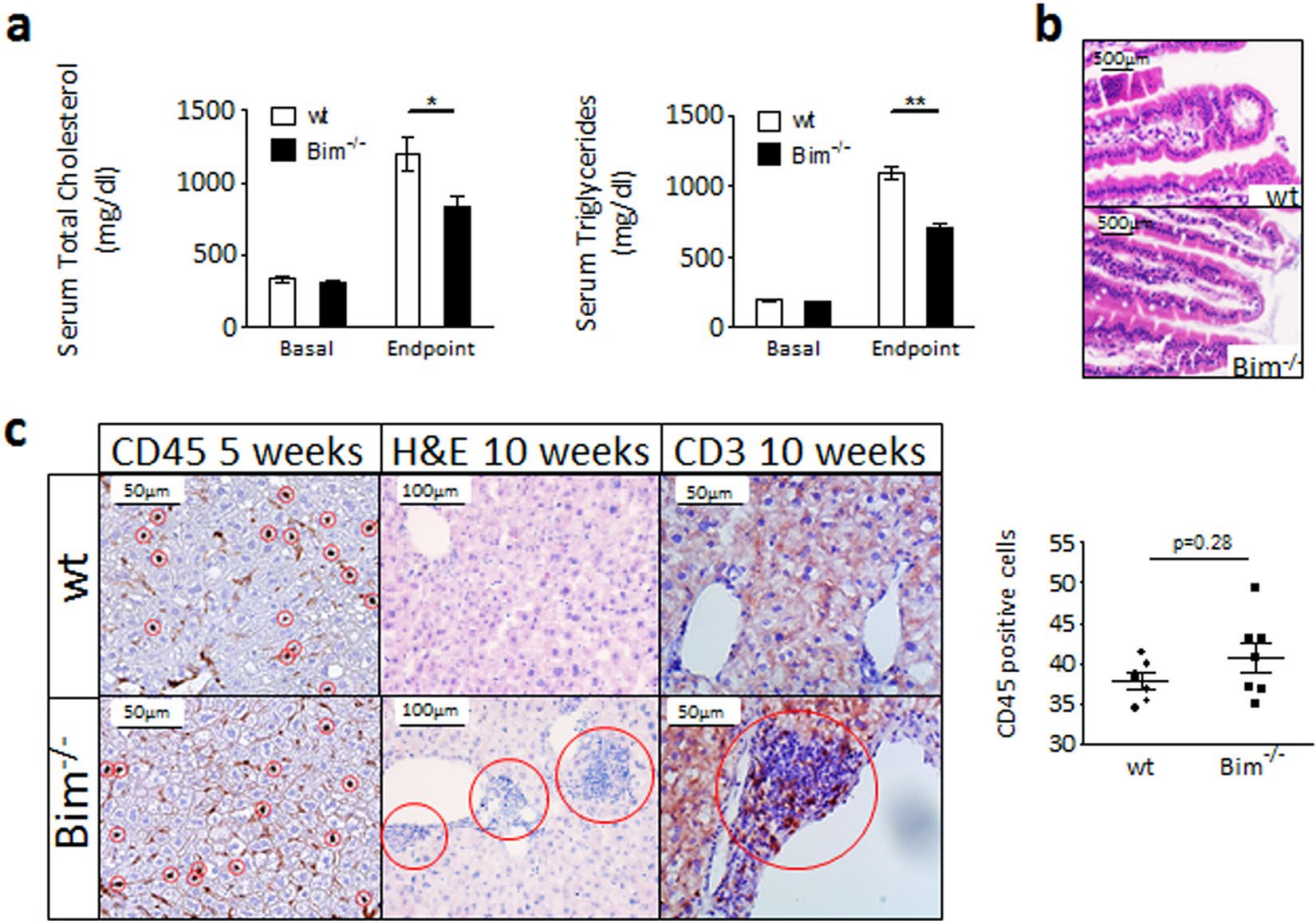
Lower cholesterol levels and liver infiltrates in *bim* ^−/−^ chimeric mice. (**a**) Cholesterol and Triglyceride levels in serum of wt and bim^−/−^ chimeric mice after before (Basal) and after 10 weeks of WTD (Endpoint). N = 12. Mean + SEM are indicated. *p < 0.05, **p < 0.01 (**b**) Representative images of H&E stained wt and bim^−/−^ duodena show intacρt villi. (**c**) Liver sections of wt and bim^−/−^ chimeric mice were analyzed for leukocyte infiltration at 5 weeks WTD (brown CD45 staining on left panels, CD45+ cells encircled in red were quantified as is shown by the graph). After 10 weeks WTD, infiltrates are clearly visible on H&E stainings (middle panels, red circles) and contain high amounts of T cells (brown CD3 staining on right panels).

Intrigued by the surprising lack of effects of bim deficiency on atherogenesis despite the overall proatherogenic phenotype of *bim* ^−/−^ mice, we zoomed in on confounders which could have masked the overt autoimmune responses in these mice. Lipids, and LDL-cholesterol in particular, are crucial in creating the proinflammatory environment critical to the development of atherosclerosis. After 10 weeks of WTD, we observed significantly reduced plasma levels of cholesterol and triglycerides (Fig. 6a). A role for (hematopoietic) leukocyte Bim in cholesterol homeostasis has not been described so far. This could be a consequence of an alerted autoimmune state interfering either at the level of cholesterol absorption in duodenum, or of its production by liver. However, body weights were unchanged, and pathological examination did not reveal any signs of autoimmune or inflammatory damage in duodena of wt and *bim* ^−/−^ chimeras, with both groups showing structurally intact villi (Fig. 6b). After 5 weeks of WTD, liver immune cell presence did not differ between *bim* ^−/−^ chimeras and controls either (Fig. 6c left panels and graph). At 10 weeks of WTD however, we noticed the overt presence of cellular infiltrates containing large amounts of T cells in livers of *bim* ^−/−^ but not wt mice (Fig. 6c, middle and right panels). Our data thus suggest that the pro-inflammatory effects of Bim deficiency are counteracted at least in part by lipid lowering, which may be due to progressive steatohepatitis compromising crucial lipid metabolic functions.

## Discussion

Bim is essential for apoptosis of various leukocyte subsets, including T and B cells, dendritic cells, macrophages and granulocytes^14, 15, 21^. These cell types are all shown to be present in atherosclerotic lesions and to contribute to disease development. In addition, Bim specifically deletes autoreactive T and B cells^18, 22^, and atherosclerosis is a disease with clear autoimmune features^23–25^. Therefore, a role for Bim in lesional leukocyte apoptosis and (autoimmune-induced) atherogenesis may be anticipated. Here we show that hematopoietic Bim deficiency impacts on inflammatory status, an effect that is counteracted by an unexpected lowering of plasma cholesterol levels and a dampening of the WTD-induced monocytosis.

Absence of Bim decreased spontaneous apoptosis of bone marrow derived macrophages as well as apoptosis induced by growth factor withdrawal. These results are consistent with previous studies showing decreased apoptosis in Bim deficient T cells^14^, DCs^21^ and granulocytes^15^ in response to various stimuli. In addition we identify Bim as a regulator of OxLDL induced apoptosis of macrophages. However, while spleen apoptotic cell content was decreased in *bim* ^−/−^ mice as expected, we did not find any differences in apoptosis of atherosclerotic lesion cells, predominantly representing macrophages and foam cells. Given the scarce T-cell presence in atherosclerotic lesions^26^, apoptosis of this subset and hence Bim deficiency associated changes herein are not detectable. Further analysis into the functionality of *bim* ^−/−^ macrophages did not reveal striking changes in polarization, though we did observe a decrease in efferocytosis in *bim* ^−/−^ macrophages. One could speculate that the combination of increased survival of *bim* ^−/−^ (plaque – thus exposed to oxLDL and other pro-apoptotic stimuli) macrophages with their decreased efferocytosis capacity leads to a null-effect on overall plaque apoptosis. In addition, Ly6C^high^ inflammatory monocytes were significantly reduced in *bim* ^−/−^ mice. Inflammatory monocytes are important contributors to plaque development and macrophage content^27^, and a lower influx of Ly6C^high^ monocytes in *bim* ^−/−^ atheromas could further temper the pro-atherogenic WTD-induced phenotype.

The most profound consequences of Bim deficiency following BMT were observed on T cell homeostasis. Lymphocytes are importantly involved in regulating immune responses in atherosclerotic lesions^26, 28^. In keeping with previous studies^14^ we showed markedly elevated circulating lymphocyte levels and splenomegaly in *bim* ^−/−^ transplanted *ldlr* ^−/−^. As suggested by Bouillet and Hildeman and colleagues lymphocytosis and splenomegaly may be caused by impaired apoptosis of leukocytes, in particular of autoreactive thymocytes and activated T cells, for which Bim has been demonstrated to be essential^16, 17^. In agreement with the latter, both CD4^+^ and CD8^+^ splenic T cells were more activated in *bim* ^−/−^ recipients than in control mice, and we observed a clear shift towards a Th1 immune profile. The elevated T cell numbers were also reflected in increased plaque T cell content indicating enhanced infiltration from circulation into the lesion. In addition to these effects on T cell immunity, Bim deficiency significantly raised B cell numbers, potentially modifying the humoral response in *bim* ^−/−^ mice. Taken together, our data shows that *bim* ^−/−^ leukocytes affect both components of the adaptive immune system in *ldlr* ^−/−^ mice.

Bim was previously reported to be necessary for apoptosis of autoreactive B cells^18^ and to prevent T-cell dependent autoimmunity^14^. Atherosclerosis is currently viewed as a lipid driven inflammatory process with features of autoimmune disease^24–26^, implicating T cell responses to auto-antigens, such as oxLDL and heat shock proteins (HSP)^25^. In fact, oxLDL specific antibodies have been detected in atherosclerosis-prone *ApoE* ^−/−^ mice^29^, in human and rabbit serum and in atherosclerotic lesions^30^. Moreover, a subset of T cells present in human lesions was demonstrated to be oxLDL specific^31^. Concordant with Bim’s role in the control of autoreactive B cell formation, we observed markedly elevated oxLDL antibody levels in serum of *bim* ^−/−^ mice. In addition, total immunoglobulin deposition in atherosclerotic lesions of *bim* ^−/−^ mice was dramatically increased. While previously B cells were considered to be atheroprotective^32–35^, more recent insights point however to divergent roles in atherosclerosis^36, 37^. B2 cells, precursors of IgG producing plasma cells, are in fact proatherogenic^38^. The observed increase in serum anti-oxLDL antibody titers in *bim* ^−/−^ mice in the present study is mainly due to elevated anti-oxLDL IgG1 levels, whereas anti-oxLDL natural antibody titers (IgM), elaborated by atheroprotective B1 cells, were not affected. This clearly points to an involvement of the proatherogenic B2 cell subset, which indeed was expanded in *bim* ^−/−^ mice. Anti-oxLDL IgG1 antibodies induce proinflammatory signaling upon binding to FcγRIII^39^, provided the activating FcγRIII receptors outnumber the inhibitory FcγRIIB molecules^40^. Inflammatory monocytes highly express FcγRIII, infiltrate the atherosclerotic plaque and become loaden with oxLDL, and are thus a likely target for the anti-oxLDL IgG1 antibodies in the *bim* ^−/−^ mice.

Despite marked effects on T cell levels and characteristics as well as on the humoral immune response, leukocyte Bim deficiency in *ldlr* ^−/−^ mice did not alter plaque size or composition at an early as well as more advanced stage of disease development, apart from increased lesional T cell and immune complex accumulation. In contrast to our results, *FcγRIIB* ^−/−^ bone marrow transplanted *ldlr* ^−/−^ mice^3^ display a systemic autoreactive phenotype with splenomegaly, increased circulating autoantibodies and large lesional immunoglobulin complexes, accompanied by aggravated lesion development. Similarly to Bim deficient mice, the *FcγRIIB* ^−/−^ *ldlr* ^−/−^ chimeras showed reduced plasma cholesterol and TG levels^3^. Hence the discrepant outcomes of this study and ours is probably owing to the much stronger autoimmune phenotype of the FcγRIIB compared to the Bim deletion model. However, recent work by Ludwinksi *et al*. shows that Bim can trigger the activation of mature autoreactive T cells, through the calcium/NFAT pathway. This explains why *Bim* ^−/−^ bone marrow transplanted mice are protected against autoimmunity in an EAE and diabetes model^41^. Accordingly, activation of atherosclerotic antigen-specific T cells, having escaped thymic selection, might be compromised by the Bim deficiency in our model, explaining the mild plaque development. Moreover, the fact that two independent autoimmune models on a similar background (*FcγRIIB* ^−/−^ and *Bim* ^−/−^ on *ldlr* ^−/−^) both lead to substantial decreases in serum lipids, suggests a shared molecular basis, linking autoimmunity to cholesterol metabolism. As we did not detect any structural damage to the villi of *bim* ^−/−^ duodena, it is unlikely that reduced intestinal cholesterol uptake is accountable for the Bim deficiency associated lipid lowering. In contrast, *bim* ^−/−^ livers displayed progressive leukocyte infiltrates highly enriched in T cells. Hepatocytes have the ability to prime naïve T cells, and the survival of liver-residing T cells is largely dependent on Bim^42^. The liver functions as a master regulator of cholesterol metabolism, and chronic steatohepatitis could conceivably lead to disturbances of lipid homeostasis in blood. Cholesterol homeostasis has already been linked to the chronic inflammatory status^43^, and in particular dendritic cell targeted interventions were repeatedly shown to alter plasma cholesterol levels^44^. It remains to be determined how hepatic T-cell inflammation contributes to the attenuated hyperlipidemic response to Western Type Diet. Nevertheless, lower plasma lipid levels in *bim* ^−/−^ animals will undoubtedly contribute to a less atherogenic environment.

In conclusion, we show here that leukocyte Bim deficiency in *ldlr* ^−/−^ mice results in increased activated T-cell content in circulation, lymphoid organs and atherosclerotic lesions, in increased levels of autoreactive antibodies directed against oxidized LDL in circulation and in substantial immunoglobulin deposition in atherosclerotic lesions. Collectively however, these proatherogenic effects of leukocyte Bim deficiency are most likely counterbalanced by a lowering of the Ly6C^high^ monocytosis accompanied by a surprising reduction in serum lipid levels, leaving atherosclerosis development unaffected.

## Methods

### Animal work and bone marrow transplantation experiment

All animal work was approved by regulatory authority of Leiden and Maastricht and performed in compliance with the Dutch government guidelines. Pure C57Bl6 *Ldlr* ^−/−^ mice (backcrossed at least 10 generations) were obtained from the local animal breeding facility. C57Bl6 *bim* ^−/−^ mice for the 10 weeks WTD study were a kind gift from the Department of Biochemistry, Biosciences Research Institute, the University College Cork, Ireland and had been backcrossed at least 6 generations. For the 5 weeks WTD study, frozen pure C57Bl6 *bim* ^−/−^ (#JR 4525) and wt control bone marrow (#JR 664) was obtained from Jackson Laboratories, Maine. Male *ldlr* ^−/−^ mice (n = 38) were housed in sterile individual ventilated cages with food and water *ad libitum*. The drinking water was supplied with antibiotics (83 mg/l ciprofloxacin and 67 mg/l Polymixin B) and 5 g/l sugar. The mice were exposed to a single dose of 9 Gy total body irradiation (0.19 Gy/min, 200 kV, 4 mA) using an Andrex Smart 225 Röntgen source (YXLON International) one day before transplantation. Bone marrow was extracted from femurs and tibia of male *bim* ^−/−^ and wild-type (wt) littermates. Irradiated *ldlr* ^−/−^ mice received either 5 × 10^6^ *bim* ^−/−^ bone marrow cells or 5 × 10^6^ wt bone marrow cells via tail vein injection. After a recovery period of six weeks diet was changed from normal chow (RM3, Special Diet Services) to Western type diet (WTD) containing 0.25% cholesterol and 15% cacao butter (Diet W, Special Diet Services) for an additional five or ten weeks.

### Blood cell analysis and flow cytometry

Blood samples were taken by tail bleeding before bone marrow transplantation (BMT) and before and after start of Western type diet feeding and at the time of sacrifice. Before start of the WTD, DNA was isolated from the blood tail vein sample using the Qiagen QIAamp® DNA Micro Kit according to manufacturer’s instructions. Chimerism was determined by quantitative real-time PCR using primers for LDLr (only present in the donor-derived cells, forward primer: 5′- GCT GCA ACT CAT CCA TAT GCA -3′, reverse primer: 5′- GGA GTT GTT GAC CTC GAC TCT AGA G -3′) and P50 (present in donor and recipient cells, forward primer: 5′- AAC CTG GGA ATA CTT CAT GTG ACT AA -3′, reverse primer: 5′ - GCA CCA GAA GTC CAG GAT TAT AGC -3′) and was 95,9% with a minimum of 88,5% for wt transplanted mice versus 96,3% with a minimum of 88,4% for *bim* ^−/−^ transplanted mice. *Bim* ^−/−^ genotype was confirmed by PCR using primers 5′-CATTCTCGTAAGTCCGAGTCT-3′ (forward), 5′-GTGCTAACTGAAACCAGATTAG-3′ (reverse, specific for wt allele) and 5′-CTCAGTCCATTCATCAACAG-3′(reverse, specific for deleted allele).

At sacrifice, mice were anesthesized and spleen was removed, gently dissociated through a 70 µm cell strainer (Greiner), treated with erylysis buffer (8.4 g NH_4_Cl, 0.84 g NaHCO_3_ in 1 l PBS) and stained for total leukocytes (CD45^+^, BioLegend), total T cells (CD3^+^, eBioscience), T helper cells (CD4^+^, BD Bioscience), cytotoxic T cells (CD8α^+^, BD Bioscience), B cells (CD19^+^, eBioscience), B1 cells (CD5^+^, eBioscience), B2 cells (B220^+^, BD and CD5^-^, eBioscience), NK cells (CD3^−^ NK1.1^+^, BD Bioscience) monocytes (CD11b^high^ Ly6G^low^, BD Bioscience) and neutrophils (CD11b^high^ Ly6G^high^, BD Bioscience). Activated T cells were stained with antibodies against CD69 and CD71 (eBioscience). IFNγ and IL4 producing T cells were quantified performing intracellular staining against IFNγ or IL4 (both eBioscience) after overnight PMA (50 ng/ml)/ionomycin (1 µg/ml) *in vitro* stimulation of isolated splenocytes. *In vivo* proliferation was assessed using the APC BrdU Flow kit (BD) according to the manufacturer’s instructions. Regulatory T cells were stained on bone marrow isolated at sacrifice after 5 weeks of WTD with FoxP3 intracellular staining kit (eBioscience) according to the manufacturer’s instructions. Absolute cell numbers in blood were calculated by use of Trucount tubes (BD). Absolute counts of other percentage-based *in vivo* flow cytometry plots throughout the paper are given in Sup. Fig. 4. All flow cytometry analysis was performed on a BDCanto II (BD Bioscience) using FACS Diva Analysis Software vs6.

### Tissue harvesting and analysis

Eleven (early atherogenesis, n = 7) or sixteen weeks (intermediate atherogenesis, n = 12) after BMT, mice received intraperitoneal injections with BrdU 24 and 12 hrs before sacrifice (0.8 mg/mouse). Next day, mice were euthanized by i.p. administration of an overdose of pentobarbital (115 mg/kg), blood was taken by left ventricular puncture, and mice were perfused with PBS after which heart, aorta, spleen, thymus, mediastinal lymph nodes, duodenum and liver were isolated and either further processed for flow cytometry, stored in 4% formaldehyde solution or snap-frozen in liquid nitrogen and stored at −80 °C. Cryosections were prepared of aortic valves, spleen and liver and stained with hematoxylin and eosin (HE) and/or Oil Red ‘O. Paraffin sections were prepared of liver and duodena and stained with HE and for the presence of leukocytes (CD45, BD). The descending aorta was cut open longitudinally for *en face* analysis after staining with Oil Red ‘O. Immunohistochemistry was performed for macrophage (MOMA-2, Sigma) and T cell (CD3, Immunologic) content in aortic root lesion, liver and/or spleen sections. Apoptotic cell content after 5 weeks of WTD was quantified by cleaved caspase 3 staining (Cell Signaling). Apoptotic cell content after 10 weeks WTD was quantified using terminal deoxytransferase dUTP nick-end labeling (TUNEL) kit (Roche Diagnostics) and presence of immunoglobulins using FITC labeled rabbit anti-mouse Ig (DakoCytomation). Lesion size and tissue morphology was analyzed using Leica image analysis system, consisting of a Leica DMRE microscope with camera and Leica Qwin Imaging software (Leica Ltd). Fluorescent immunohistochemistry was analyzed on a Nikon Eclipse E600 using ImagePro 4.5 software.

### Detection of auto-antibodies by ELISA

Presence of anti-dsDNA autoantibodies was confirmed in collaboration with Dr. J. van der Vlag (Radboud University Medical Center, Nijmegen, Netherlands) using a peptide ELISA technique extensively described in Dieker *et al*.^45^. For quantification of anti-oxLDL autoantibodies, an EIA/RIA high binding 96-well plate (Corning) was coated with Ox-LDL (5 μg/ml) in a 50 mM Na_2_CO_3_/NaHCO_3_ coating buffer (pH 9.6). IgM, IgG1 and IgG2a antibodies against Ox-LDL in serum were measured using an enzyme-linked immunosorbent assay (ELISA) Ig detection kit (Zymed Laboratories) according to the manufacturer’s protocol.

### Macrophage apoptosis

Femurs and tibia were flushed with phosphate buffered saline (PBS) to isolate bone marrow. A single cell suspension was obtained by passing the bone marrow through a 70 µm nylon cell strainer (BD Falcon). Bone marrow cells were differentiated into macrophages by culturing in 70% RPMI, supplemented with 20% FCS, glutamine, pyruvate, penicillin/streptomycin and non-essential amino acids, and 30% M-CSF conditioned DMEM (obtained from L929 cells) for 7 days. Bone marrow derived macrophages (BMDM) were stimulated with 40 µg/ml ox-LDL, cultured without growth factors (30% M-CSF conditioned DMEM) or in control medium for 24 hours. The macrophages were detached with accutase (PAA Laboratories GmbH), stained with Annexin V (ImmunoTools) and propidium iodide (Sigma) and subsequently analyzed by flow cytometry using a FACSCalibur with CellQuest software (BD Biosciences).

### Cholesterol and triglyceride levels

Blood samples were taken by tail bleeding before bone marrow transplantation (BMT) and before and after start of Western type diet feeding and at the time of sacrifice. Total cholesterol and triglyceride content was measured spectrophotometrically in serum using enzymatic procedures (Roche Diagnostics).

### Real time PCR on sorted cells

Bone marrow was isolated by flushing femurs of wt and *bim* ^−/−^ mice after 5 weeks of WTD at sacrifice. Mature CD4^+^ T cells were present in both wt and *bim* ^−/−^ bone marrow in numbers consistent with literature^46^. CD4^+^ T cells were sorted as not dead, CD45^+^ (BioLegend), forward scatter low, CD19^−^ (eBioscience), NK1.1^−^ (BD Bioscience), Ly6G^−^ (BD Bioscience) CD8α^−^ (BD Bioscience), CD4^+^ (BD Bioscience). BMDM precursor cells were sorted as not dead, CD45^+^, forward scatter low, CD19^−^, NK1.1^−^, Ly6G^−^, CD8α^−^ and CD4^−^, pooled together with not dead, CD45^+^, forward scatter high, CD19^−^, NK1.1^−^, Ly6G^−^ and not dead, CD45^+^, CD19^+^, NK1.1^+^, Ly6G^+^ cells. Sorting was done on a BD Aria 1 machine. Sorted CD4^+^ T cells were *in vitro* stimulated with PMA (50 ng/ml)/ionomycin (1 µg/ml) overnight before lysis with TRIzol®. BMDM precursors were differentiated into macrophages as described above and stimulated for 3 hours with LPS (50 ng/ml) or for 6 hours with IL4 (50 ng/ml) before lysis with TRIzol®. RNA was prepared using the Qiagen Micro RNAeasy Kit following manufacturer’s instructions. cDNA preparation was done with the iScript cDNA Synthesis Kit (BioRad). Real time PCRs were performed on a BioRad CFX-96 Cycler using its software for analysis. Primer pairs used were IL 10 (fw: 5′- TTT GAA TTC CCT GGG TGA GAA – 3′, rv: 5′- CTC CAC TGC CTT GCT CTT ATT TTC - 3′), IFNγ (fw: 5′- ATC TGG AGG AAC TGG CAA AA - 3′, rv: 5′- TTC AAG ACT TCA AAG AGT CTG AGG TA - 3′), IL6 (fw: 5′- CTG CAA GAG ACT TCC ATC CAG TT - 3′, rv: 5 ‘- GAA GTA GGG AAG GCC GTG G - 3′), TNFα (fw: 5′- CAT CTT CTC AAA ATT CGA GTG ACA A – 3′, rv: 5′- TGG GAG TAG ACA AGG TAC AAC CC - 3′), Mannose Receptor (fw: 5′-TGC CAA AAA TTA TTG ATC CTG TAA CT - 3′, rv: 5′- CGC CGG CAC CTA TCA CA - 3′), Fizz1 (fw: 5′- CTG CCC TGC TGG GAT GAC - 3′, rv: 5′- TCC ACT CTG GAT CTC CCA AGA - 3′) and YM1 (fw: 5′- TGG CCC ACC AGG AAA GTA CA - 3′, rv: 5′- CAG TGG CTC CTT CAT TCA GAA A - 3′). GAPDH (fw: 5′- CAA CTC ACT CAA GAT TGT CAG CAA – 3′, rv: 5′ - TGG CAG TGA TGG CAT GGA – 3′) was used as housekeeping gene.

### Efferocytosis assay

Jurkat T were rendered apoptotic by exposure to UV-B light, labeled with home-made Annexin-V-pHrodo Green (2,5 ng/ml) and fed in a 3:1 ratio to Hoechst-labeled BMDMs derived from sorted bone marrow as described above. After 45 min, BMDMs were washed and imaged using a BD Pathway 855 High-Content Imager with a 10x magnification. Image analysis was done using Attovision Software and image data was further processed using DIVA software.

### Statistical analysis

All data is presented as mean ± SEM. Data was processed using GraphPad Prism 5 (Graph Pad Software Inc., San Diego, CA, USA). Individual groups of normally distributed data were analyzed with a Student’s *t*-test, otherwise a non-parametric Mann-Whitney *U* test was used. Data containing more than two groups was analyzed with 1-way ANOVA (when showing a Gaussian distribution) or the non-parametric Kruskal-Wallis test, and results were corrected for multiple testing. Differences were considered significant if p < 0.05 (denoted as *p < 0.05, **p < 0.01, ***p < 0.001).

## Electronic supplementary material

Supplementary Material
