# Supplementary material for: Leukocyte Bim deficiency does not impact atherogenesis in ldlr−/− mice, despite a pronounced induction of autoimmune inflammation
Source: Sci Rep. 2017 Jun 8;7:3086. doi: 10.1038/s41598-017-02771-4 (PMC5465223; doi:10.1038/s41598-017-02771-4)
Supplement: Supplementary file 1 — Supplementary Material [file 41598_2017_2771_MOESM1_ESM.pdf]

Leukocyte Bim deficiency does not impact atherogenesis in *ldlr*$^{-/-}$ mice, despite a pronounced induction of autoimmune inflammation

[*]Lieve Temmerman [1,†], Marijke M Westra [2,†], Ilze Bot [2], Bart J M van Vlijmen [3], Niek Van Bree [1], Martine Bot [2], Kim L L Habets [2], Tom G H Keulers [4], Johan van der Vlag [5], Thomas G Cotter [6], Theo J C van Berkel [2] and Erik A L Biessen [1,7]

[†] These authors contributed equally

[1] Experimental Vascular Pathology, Department of Pathology, Cardiovascular Research Institute Maastricht, Maastricht University, Maastricht, the Netherlands

[2] Division of Biopharmaceutics, Leiden Amsterdam Centre for Drug Research, Leiden University, Leiden, the Netherlands

[3] Department of Thrombosis Hemostasis, Leiden University Medical Centre, Leiden, the Netherlands

[4]Department of Radiotherapy (MAASTRO)/GROW, School for Developmental Biology and Oncology, Maastricht University, Maastricht, The Netherlands

[5] Department of Nephrology, Radboud University Medical Center, Nijmegen, the Netherlands

[6] Cell Development and Disease Laboratory, Department of Biochemistry, Biosciences Research Institute, University College Cork, Cork, Ireland

[7]Institute for Molecular Cardiovascular Research (IMCAR), University Hospital RWTH Aachen, Germany

[*] Corresponding author: Lieve Temmerman, PhD
Experimental Vascular Pathology
Department of Pathology
Cardiovascular Research Institute Maastricht
Maastricht University
Maastricht, the Netherlands
Phone: +31 (0)43 387 46 33
Fax: +31 (0)43 387 66 13
lieve.temmerman@mumc.nl

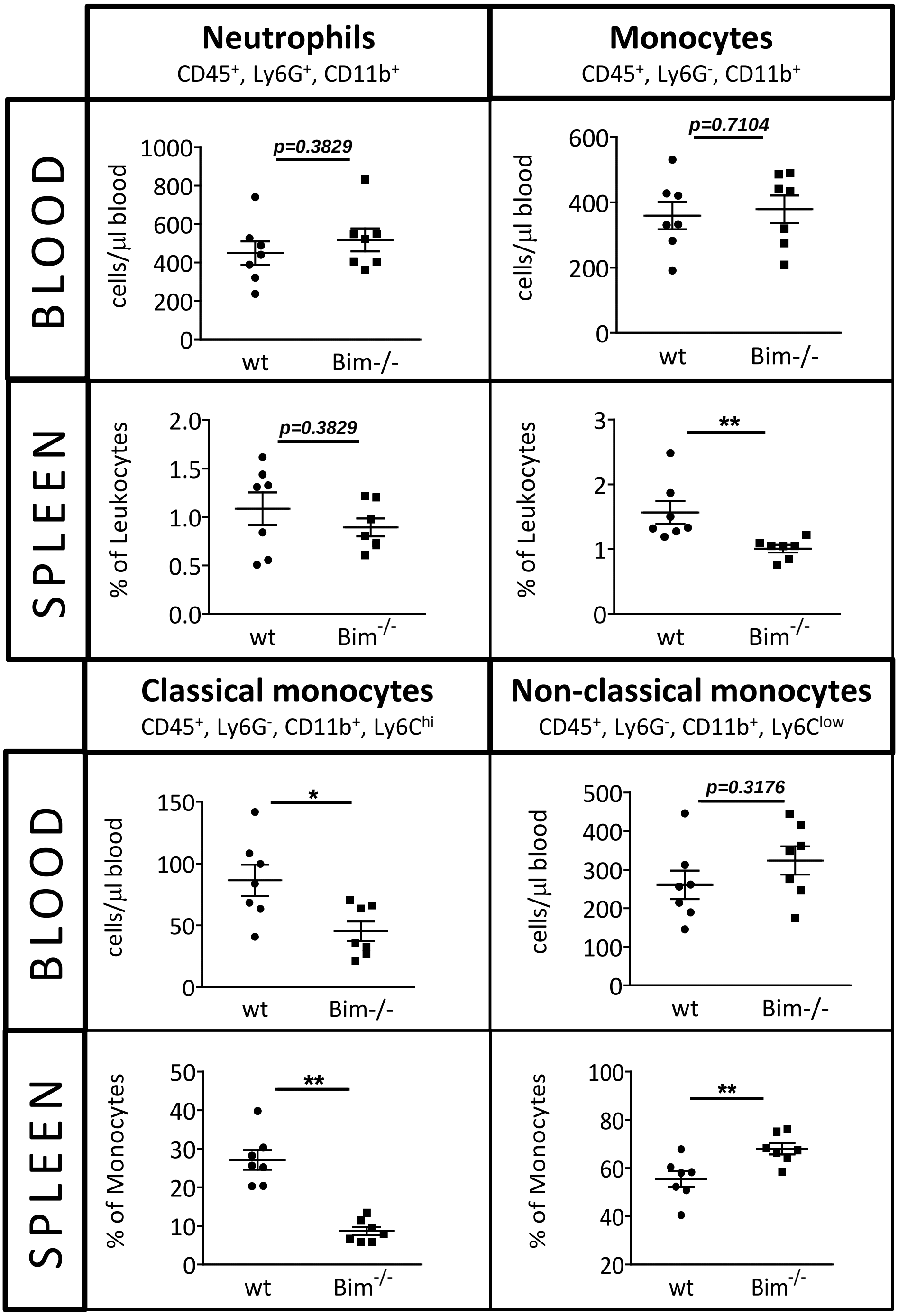

Supplementary Figure 1. Innate immune response in wt and *bim*$^{-/-}$ chimeric mice

Flow cytometry measurements to quantify neutrophils and monocytes in blood (TruCount) and spleen after 5 weeks of WTD (n=7). Neutrophils were defined as $CD45^+$, $Ly6G^+$, $CD11b^+$. Monocytes were defined as $CD45^+$, $Ly6G^-$, $CD11b^{hi}$ and further subdivided in a classical $Ly6C^{hi}$ and non-classical $Ly6C^{low}$ subset. Data is presented as mean ± SEM. *: $p<0.05$, **: $p<0.01$.

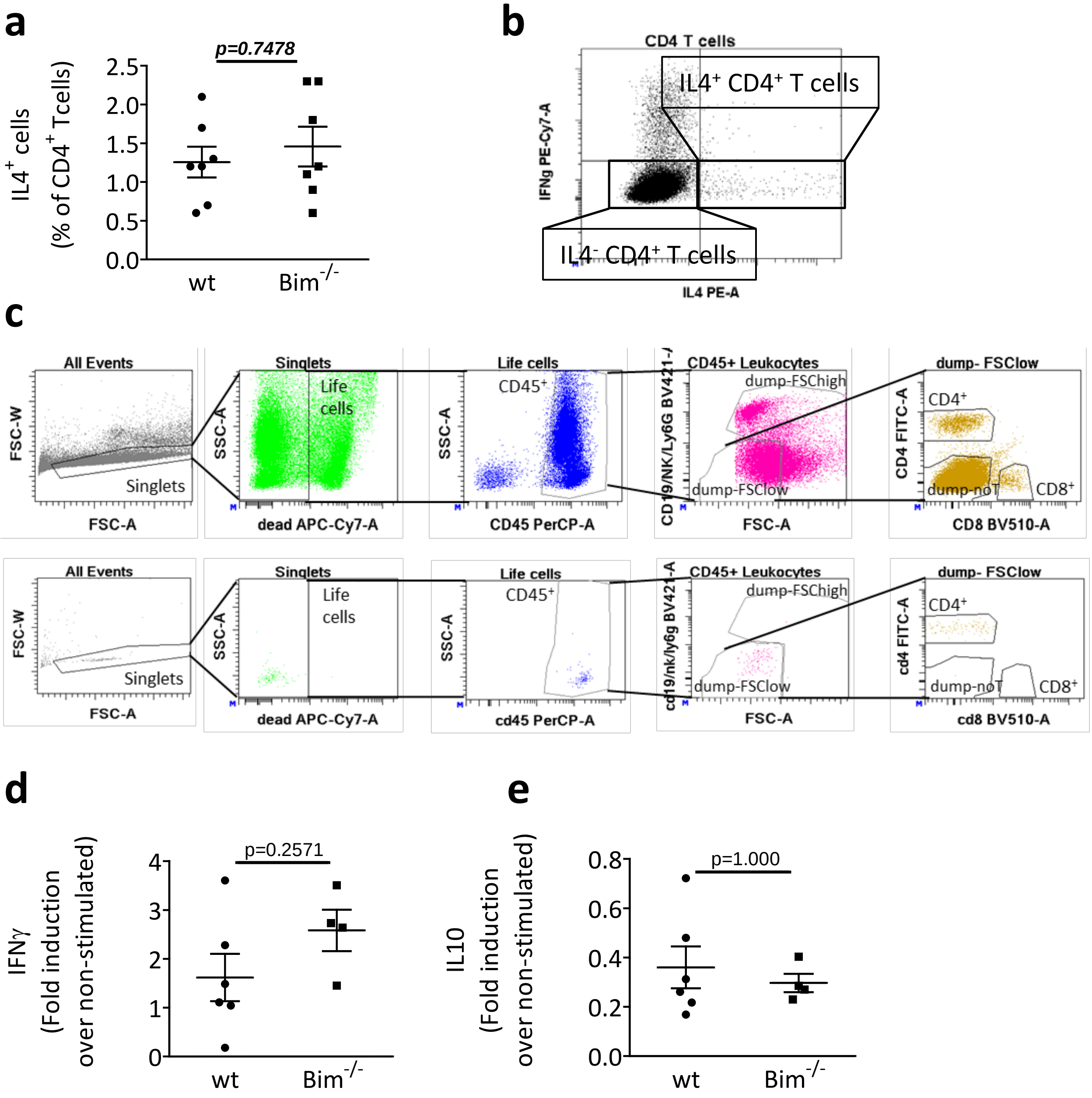

Supplementary Figure 2. Cytokine profile of wt and *bim*$^{-/-}$ CD4^{+}T cells

(**a**) Wt and *bim*$^{-/-}$ splenocytes were harvested after 5 weeks of WTD, stimulated *in vitro* with PMA and ionomycin and IL4 production in T cells was quantified using flow cytometry (n=7). (**b**) Gatings for the IL4 positive cells quantified in (a). (**c**) CD4^{+} T cells were harvested from bone marrow of wt and *bim*$^{-/-}$ chimeric mice after 5 weeks of WTD by FACS sorting. Sorting gate strategy (upper panel) is shown. Recovered CD4^{+} T cells were >99% pure (lower panel). (**d**) CD4^{+} T cells sorted in (c) were stimulated *in vitro* with PMA and ionomycin. Real-time PCR results show expression levels of IFNγ compared to expression levels in unstimulated control cells. (n=4-6). (**e**) CD4^{+} T cells sorted in (c) were stimulated *in vitro* with PMA and ionomycin. Real-time PCR results show expression levels of IL10 compared to expression levels in unstimulated control cells. (n=4-6). Data is presented as mean $\pm$ SEM.

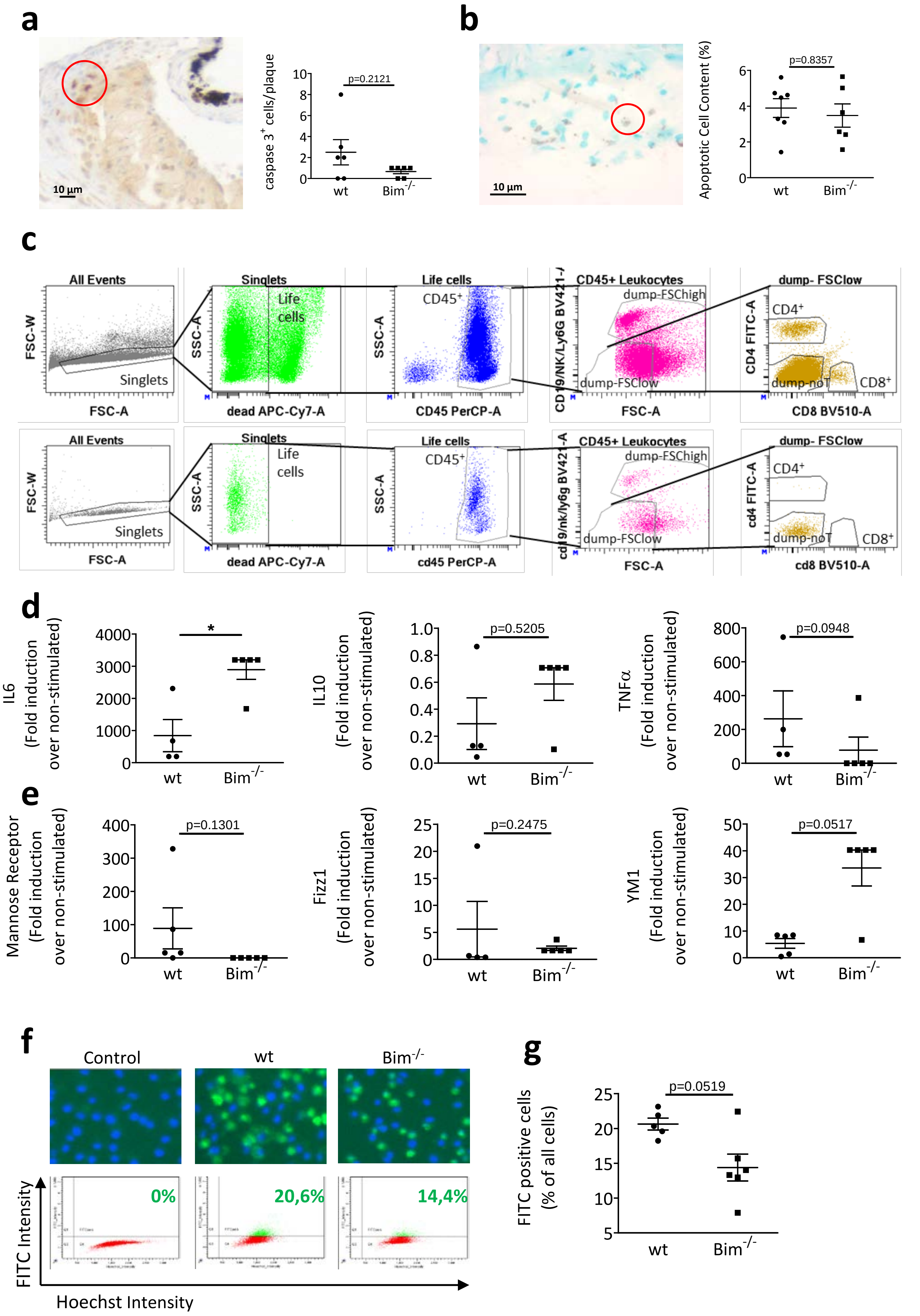
a
10 µm
caspase 3+ cells/plaque
p=0.2121
wt
Bim-/-
b
10 µm
Apoptotic Cell Content (%)
p=0.8357
wt
Bim-/-
c
All Events
Singlets
FSC-W
FSC-A
Life cells
SSC-A
dead APC-Cy7-A
CD45+
CD45 PerCP-A
CD45+ Leukocytes
dump-FSChigh
dump-FSClow
FSC-A
dump- FSClow
CD4+
CD8+
dump-noT
CD4 FITC-A
CD8 BV510-A
d
IL6 (Fold induction over non-stimulated)
*
IL10 (Fold induction over non-stimulated)
p=0.5205
TNFα (Fold induction over non-stimulated)
p=0.0948
e
Mannose Receptor (Fold induction over non-stimulated)
p=0.1301
Fizz1 (Fold induction over non-stimulated)
p=0.2475
YM1 (Fold induction over non-stimulated)
p=0.0517
f
Control
wt
Bim-/-
0%
20,6%
14,4%
FITC Intensity
Hoechst Intensity
g
FITC positive cells (% of all cells)
p=0.0519
wt
Bim-/-

Supplementary Figure 3. No difference in apoptosis in aortic roots of wt and *bim*$^{-/-}$ chimeric mice

(**a**) Representative picture of aortic root section stained for cleaved caspase 3 (brown, nuclear) after 5 weeks WTD. Red circle marks positively stained cells. Data comparing cleaved caspase 3 positive cells in wt and *bim*$^{-/-}$ chimeric plaques (n=6) is shown as mean $\pm$ SEM. (**b**) Representative picture of TUNEL-stained aortic root section (brown, nuclear) after 10 weeks WTD. Red circle marks positively stained cell. Data comparing TUNEL positive cell content in wt and *bim*$^{-/-}$ chimeric mice (n=6-7) is shown as mean $\pm$ SEM. (**c**) BMDM precursor cells were harvested from bone marrow of wt and *bim*$^{-/-}$ chimeric mice after 5 weeks of WTD by FACS sorting. Sorting gate strategy (upper panel) is shown. Recovery of sorted cells (dump-FSChigh + dump-noT) contained less than 1% contamination by other populations (lower panel). (**d-e**) BMDM precursor cells sorted in (c) were differentiated into BMDMs for one week and stimulated with LPS (d) or IL-4 (e) to obtain M1 and M2 like cells respectively. Real-time PCR results show expression levels of M1 cytokines IL6, IL10 and TNFα (d) and M2 cytokines Mannose Receptor, Fizz1 and YM1 (e) compared to expression levels in unstimulated control cells. (n=4-6). Data is presented as mean $\pm$ SEM. *: $p<0.05$ in Mann-Withney U Test. (**f**) Representative 10x pictures and data image analysis showing efferocytosis (% of FITC positive cells) in control BMDMs and in wt and *bim*$^{-/-}$ BMDMs exposed to apoptotic FITC-labeled Jurkats. (**g**) Mann-Withney U Test reveals no difference between wt and *bim*$^{-/-}$ BMDMs in efferocytosis. Data is presented as mean $\pm$ SEM.

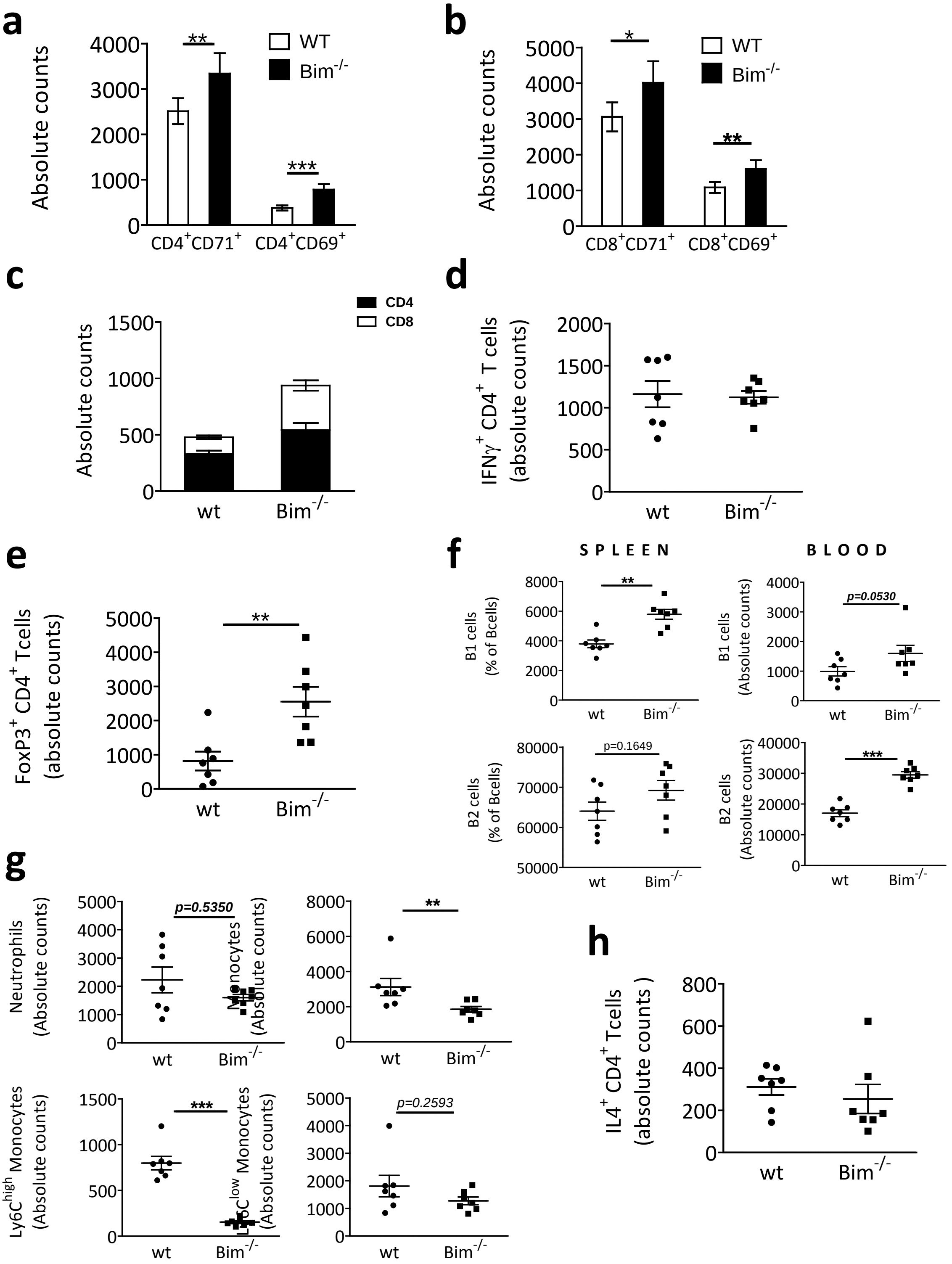

Supplementary Figure 4. Absolute counts of FACS plots

Absolute counts of cell populations are given for FACS plots throughout the manuscript. Data is presented as mean ± SEM.

(**a**) Absolute counts of activated T cell plot in Fig. 3a. (**b**) Absolute counts of activated T cell plot in Fig. 3b. (**c**) Absolute counts of T cell ratio plot in Fig. 3c. (**d**) Absolute counts of IFNγ^{+} Tcell plot in Fig. 3e. (**e**) Absolute counts of FoxP3^{+} Tcell plot in Fig. 3h. (**f**) Absolute counts of B1 and B2 cell plots in Fig. 4c. (**g**) Absolute counts of spleen neutrophil and monocytes plots in Sup. Fig. 1. (**h**) Absolute counts of IL4^{+} Tcell plot in Sup. Fig. 2a.
